# Retraction: Plexin-B1 Activates NF-κB and IL-8 to Promote a Pro-Angiogenic Response in Endothelial Cells

**DOI:** 10.1371/journal.pone.0223295

**Published:** 2019-09-26

**Authors:** The PLOS ONE Editors

Following publication of this article [1], the *PLOS ONE* Editors received notification that an internal investigation conducted by the University of Maryland, Baltimore found that in Fig 2B, the histogram with the group data for I-κB super-repressor (SR) is a duplicate of the histogram in Fig 2A for the BAY11-7085-treated cells with a change of a label, calling into question the reliability of the results. A recommendation was made to retract the article in order to correct the scientific record and ensure its integrity.

In light of the findings and recommendation of the institutional investigation, the *PLOS ONE* Editors retract this article.

PP and JRB agreed with the retraction. Y-HY, HZ, and NOB could not be reached.
